# An Integrated Approach to Addictive Behaviors: A Study on Vulnerability and Maintenance Factors

**DOI:** 10.3390/ejihpe13030039

**Published:** 2023-02-21

**Authors:** Alessio Gori, Eleonora Topino, Marco Cacioppo, Giuseppe Craparo, Adriano Schimmenti, Vincenzo Caretti

**Affiliations:** 1 Department of Health Sciences, University of Florence, Via di San Salvi 12, Pad. 26, 50135 Firenze, Italy; 2 Integrated Psychodynamic Psychotherapy Institute (IPPI), Via Ricasoli 32, 50122 Florence, Italy; 3 Department of Human Sciences, LUMSA University of Rome, Via della Traspontina, 21, 00193 Rome, Italy; 4 Faculty of Human and Social Sciences, UKE—Kore University of Enna, Cittadella Universitaria, 94100 Enna, Italy

**Keywords:** mental health, prevention, risk factors, substance abuse, substance use, behavioral addiction

## Abstract

This study aimed to explore the relationships among the variables involved in a Comprehensive Model of Addiction (CMA), which posits that the presence and severity of addictive behaviors are related to the configuration of seven psychological variables, namely childhood trauma, insecure attachment, affect dysregulation, dissociation, impulsivity, compulsiveness, and obsessiveness. A vulnerability model was proposed, in which it was suggested that affect dysregulation and complex trauma mediated the association between insecure attachment and dissociation. Furthermore, a maintenance model was elaborated, in which it was hypothesized that dissociation influenced affect dysregulation via impulsivity, compulsiveness, and obsessiveness. A clinical sample of 430 individuals with substance use disorder was involved. All participants received a DSM-5 clinical diagnosis of Substance-Related and Addictive Disorders and were recruited from the Italian National Health System. A parallel mediation emerged, confirming the vulnerability model, with complex trauma and affect dysregulation mediating the relationship between insecure attachment and dissociation. Furthermore, a mixed serial–parallel mediation described the maintenance model, where impulsiveness, compulsiveness, and obsessiveness significantly mediated the relationship between dissociation and affect dysregulation. Our findings offer a better understanding of the variables associated with addictive disorders, thus providing important indications for both treatment and preventive interventions.

## 1. Introduction

The American Society of Addiction Medicine [1] defined addiction as “a treatable, chronic medical disease involving complex interactions among brain circuits, genetics, the environment, and an individual’s life experiences. People with addiction use substances or engage in behaviors that become compulsive and often continue despite harmful consequences” (p. 2). As underlined by this definition and in line with the recent studies on this topic, it appears evident that addiction is an extremely complex disease. From a phenomenal point of view, there is a great variance in individuals’ susceptibility to addiction [2], suggesting the presence of several factors determining a greater vulnerability to this disease. Furthermore, it is well known that these disorders are chronic and recurrent [3]; thus, it is crucial to understand which factors might be responsible for the maintenance of addictions.

With this in mind, Caretti and colleagues [4] identified insecure attachment, emotion dysregulation, complex trauma, dissociation, impulsiveness, compulsiveness, and obsessiveness as the key psychological variables that may be implied in addictive disorders. These variables were included in the Comprehensive Model of Addiction (CMA) [5], which is based on the assumption that the development and maintenance of addictive behaviors in an individual can be understood based on: (a) failures in attachment relationships during childhood, which prompt maladaptive dispositions toward other relationships (attachment insecurity); (b) difficulty modulating emotions, identifying and describing feelings, using feelings as a guide for one’s behavior (emotion dysregulation); (c) distressing experiences and psychological traumatization inside and outside the family (complex trauma); (d) difficulty integrating and processing mental and bodily states (dissociation); (e) a tendency to act on the spur of the moment and without considering the potentially negative consequences (impulsiveness); (f) a tendency to enact and repeat behaviors while feeling unable to stop them; (g) a tendency to become excessively involved and preoccupied with ideas or thoughts (obsessiveness).

Gori and colleagues [5] elaborated an explanation of the relationship among these variables in gambling disorders by integrating previous scientific evidence [6,7]. Therefore, emphasis was placed on the experiences with caregivers and insecure attachment, and negative developmental environments were considered risk factors for addiction occurrence [8,9,10,11,12]. These attachment failures might represent the source of childhood traumatic experiences [13] and deficits in emotion regulation skills [14], with difficulty modulating, processing, and communicating emotions (i.e., alexithymia; [15]) as a consequence; this condition, in turn, could lead to a defensive withdrawal into dissociated mental states to cope with painful emotions [16,17,18,19,20,21], which has been associated both theoretically and empirically with addictive behaviors [22,23,24,25]. However, the escape into the temporary retreat of substance use or addictive behavior further hinders the possibility of developing regulatory skills [4] by pushing toward an impulsive and compulsive search for immediate (but not lasting) gratification and making the substance or behavior as central in the individual’s life with recurrent and persistent thoughts, therefore facilitating the perpetuation of the addiction and, ultimately, hindering the treatment [26].

### Aim and Hypotheses

The present research aimed to define, complete, and empirically test this Comprehensive Model of Addiction (CMA), previously partially conceptualized by Gori and colleagues [5] in a sample of subjects with a diagnosis of Substance-Related and Addictive Disorders. More specifically, the relationships between the variables that may contribute to vulnerability and maintenance of addiction disorder were tested by implementing two mediation models.

**Figure 1:**
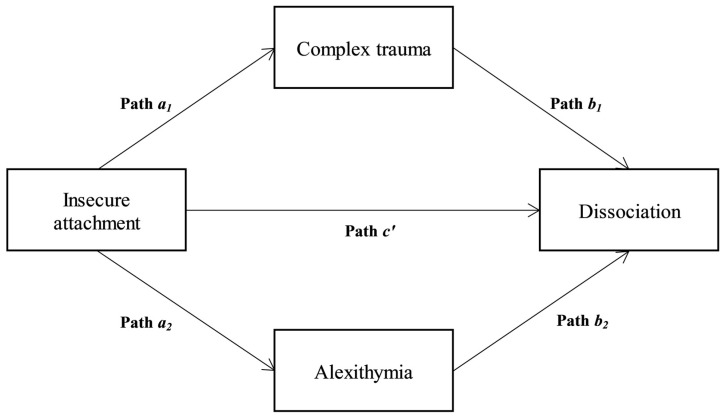
The Vulnerability model: a collateral mediation. ***Note***: The paths indicate the regression coefficients that make the model [27,28].

In the first model (vulnerability model), it was supposed that emotion dysregulation and complex trauma mediate the association between insecure attachment and dissociation (see Figure 1). In more detail, a parallel mediation model with two mediators was carried out by hypothesizing that:

**H1:**  *Insecure attachment correlates with dissociation;*

**H2:**  *Insecure attachment is related to complex trauma and affect dysregulation, the mediating variables;*

**H3:**  *Complex trauma and affect dysregulation predict dissociation;*

**H4:**  *The effect of insecure attachment on dissociation is mediated by complex trauma and alexithymia.*

**Figure 2:**
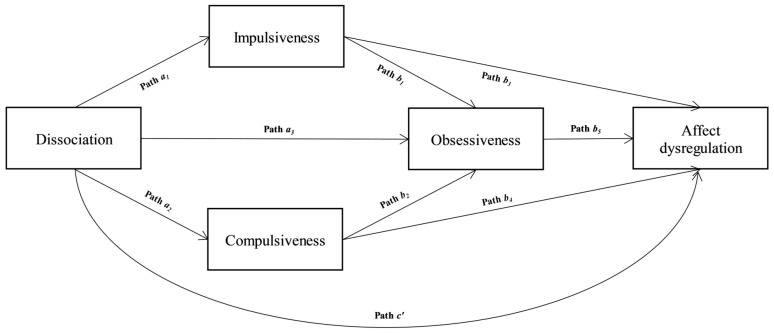
The Maintenance model: a mixed serial–parallel mediation. ***Note***: The paths indicate the regression coefficients that make the model [27,28].

Then, a second model (maintenance model) is examined, in which impulsiveness, compulsiveness, and obsessiveness are linked with dissociation and affect dysregulation. The relationship between the variables was therefore investigated by implementing a mixed serial–parallel mediation with three mediators. Specifically, it was supposed that dissociation predicts affect dysregulation, and this association is mediated by impulsiveness, compulsiveness, and obsessiveness (see Figure 2). More specifically, the hypotheses were:

**H5:**  *Dissociation correlates with affect dysregulation;*

**H6:**  *Dissociation is related to impulsiveness and compulsiveness;*

**H7:**  *Impulsiveness and compulsiveness predict obsessiveness;*

**H8:**  *The effect of dissociation on obsessiveness is mediated by impulsiveness and compulsiveness;*

**H9:**  *Impulsiveness, compulsiveness, and obsessiveness were related to affect dysregulation;*

**H10:**  *The effect of dissociation on affect dysregulation is mediated by impulsiveness, compulsiveness, and obsessiveness.*

## 2. Materials and Methods

### 2.1. Participants and Procedure

This study involved a clinical sample of 430 participants with a clinical diagnosis of “*Substance-Related and Addictive Disorders*” according to the DSM-5 criteria. They were mainly men (70%) and had an average age of 36 years (*SD* = 12.23). As shown in Table 1, most of them declared to be unemployed (34%), single (58%), and have a High School diploma (42%). Some National Health Drugs Services (Ser.D. Italy) and FeDerSerD (Italian Federation of Dependency Departments and Services Operators) provided their support and their collaboration to individuate the participants, who were recruited at the National Health System (NHS). Participation in the research was voluntary, and data were collected anonymously in a one-to-one setting. Before starting, each participant was informed about the general aim of the research and provided written informed consent. When the survey was administered, participants were still in treatment and in a detoxified state. All procedures were approved by the first author’s institutional Ethical Committee (IPPI; ethical approval number 001/2019).

### 2.2. Measures

#### 2.2.1. Addictive Behavior Questionnaire (ABQ)

The Addictive Behavior Questionnaire (ABQ) [4] is a self-report measure designed for the assessment of Substance-Related and Addictive Disorders. The first section focuses on an evaluation of the presence and frequency of the addiction disease. The second section includes: (1) the Severity Index (SI), consisting of 4 parts (substances, alcohol, gambling, internet), each allowing for the assessment of the addictive behaviors; (2) the Seven Domains Addiction Scale (7DAS), exploring 7 psychological core domains in addiction disorders (separation anxiety, affect dysregulation, somatoform and psychological dissociation, childhood traumatic experiences, impulse dyscontrol, compulsive behavior and ritualization, and obsessive thoughts), each investigated with 7 items with a 5-point Likert scale. In the present research, the 7DAS subscales were used, for which satisfactory internal consistency was found (Cronbach α ranging from 0.67 to 0.87).

#### 2.2.2. Psychological Treatment Inventory—Attachment Styles Scale (PTI-ASS)

The Psychological Treatment Inventory Attachment Styles Scale (PTI-ASS) [29] is a self-report measure designed for the assessment of adult attachment style in romantic relationships. It is a section of the Psychological Treatment Inventory [30]. The PTI-ASS consists of 22 items on a 5-point Likert scale (from 1 = “Not at All” to 5 = “A Great Deal”), grouped into 4 factors: secure, preoccupied, avoidant, and unresolved attachment styles. In the present research, a satisfactory internal consistency was found (Cronbach α ranging from 0.67 to 0.81).

#### 2.2.3. Twenty-Items Toronto Alexithymia Scale (TAS-20)

The Twenty-Items Toronto Alexithymia Scale (TAS-20) [31,32,33] is a self-report measure designed for the assessment of the level of alexithymia. It consists of 20 items scored on a 1 (=“strongly disagree”) to 5 (=“strongly agree”) Likert scale, grouped in a 3-factor structure: (1) difficulty identifying feelings and distinguishing between feelings and bodily sensations in emotional activation, (2) difficulty describing feelings, and (3) externally-oriented thinking. In this study, the total score was used, and it showed a good internal consistency (α = 0.80).

#### 2.2.4. Traumatic Experiences Checklist (TEC)

The Traumatic Experiences Checklist (TEC) [10,34] is a self-report measure designed for the assessment of 29 types of potentially traumatizing events. It consists of 29 items scored on a true-false form; for the events that occurred, the participant is also asked to rate the extent of the impact on a 5-point Likert scale (from 1 = “none” to 5 = “an extreme amount”). In this study, a total complex trauma score was used (by summing the responses of the Likert scale for each potentially traumatizing event), and it showed a good internal consistency (α = 0.81).

#### 2.2.5. Dissociative Experience Scale-II (DES-II)

The Dissociative Experiences Scale-II (DES-II) [17,35] is a self-report measure designed for the assessment of dissociative symptoms. It consists of 28 items, scored on an 11-point scale ranging from 0% (“never”) to 100% (“always”), grouped into 3 subscales: (1) dissociative amnesia; (2) absorption and imaginative involvement; (3) depersonalization-derealization. In this study, the total score was used, and it showed a good internal consistency (α = 0.94).

### 2.3. Data Analysis

The SPSS 21.0 software was used to perform the analyses. The statistical significance was set at *p* < 0.05. Descriptive statistics have been calculated, and Pearson’s *r* correlations (2-tailed type) were examined to explore the associations between the variables. Mediation analyses, a regression-based approach, were implemented to examine the hypothesized mediation models using macro-program PROCESS 3.4 applying Model 4 and Model 80 [36]. The 95% confidence interval (CI) was calculated for each regression coefficient. Finally, the statistical stability of the models was estimated by performing the bootstrapping procedure at 95% bootstrap confidence interval (CI), based on 5000 resamples: if the interval (from boot Lower Limit Confidence Interval [LLCI] to boot Upper Limit Confidence Interval [ULCI]) does not include zero, the indirect effect is considered to be statistically significant [37].

## 3. Results

The demographic characteristics of the sample are reported in Table 1. The results of correlation analyses are reported in Table 2.

Correlation analyses confirmed the hypothesized associations among the variables of interest. Results showed significant and positive associations between insecure attachment scores and alexithymia, complex trauma, and dissociation. Consistently, dissociation was also significantly and positively related to both alexithymia and complex trauma. Furthermore, affect dysregulation positively and significantly correlated with obsessiveness, compulsiveness, impulsiveness, and dissociation. Impulse dyscontrol showed significant and positive associations with both obsessiveness and dissociation. Finally, compulsiveness was positively and significantly related to both obsessiveness and dissociation.

Then, the mediation analyses were conducted (see Table 3 and Table 4).

About the first model (vulnerability model), a parallel mediation emerged. A significant total effect was shown in the association between insecure attachment and dissociation (*β* = 0.39, *p* < 0.001; LLCI = 1.268—ULCI = 1.931; **H1**). Insecure attachment was also related to complex trauma (path *a*_1_ in Figure 1; *β* = 0.36, *p* < 0.001) and alexithymia (path *a*_2_ in Figure 1; *β* = 0.29, *p* < 0.001; **H2**). The third step of this mediation model indicated that both complex trauma (path *b*_1_ in Figure 1; *β* = 0.16, *p* < 0.001) and alexithymia (path *b*_2_ in Figure 1; *β* = 0.34, *p* < 0.001; **H3**) predicted dissociation. So, the effect of insecure attachment on dissociation was reduced after controlling for complex trauma and alexithymia (path *c′* in Figure 1; *β* = 0.24, *p* < 0.001) albeit remaining significant, suggesting a partial mediation (**H4**): *R^2^* = 0.281, *F*(3, 426) = 64.878, *p* < 0.001. Finally, the bootstrapping technique confirmed the significance of the indirect effect: Boot LLCI = 0.384—Boot ULCI = 0.918.

Concerning the evaluation of the maintenance model, a mixed serial–parallel mediation was outlined. A significant total effect in the association between dissociation and affect dysregulation was shown (*β* = 0.42, *p* < 0.001; LLCI = 0.144—ULCI = 0.214; **H5**). Dissociation was related to impulsiveness (path *a*_1_ in Figure 2; *β* = 0.541, *p* < 0.001) and compulsiveness (path *a*_2_ in Figure 2; *β* = 0.45, *p* < 0.001; **H6**). Furthermore, dissociation was associated with obsessiveness both directly (path *a*_3_ in Figure 2; *β* = 0.07, *p* < 0.05; **H8**) and indirectly through the mediation of impulsiveness (path *b*_1_ in Figure 2; *β* = 0.48, *p* < 0.001) and compulsiveness (path *b*_2_ in Figure 2; *β* = 0.35, *p* < 0.001; **H7**). The next step of this mediation model indicated that obsessiveness (path *b*_5_ in Figure 2; *β* = 0.45, *p* < 0.001), impulsiveness (path *b*_3_ in Figure 2; *β* = 0.20, *p* < 0.001), and compulsiveness (path *b*_4_ in Figure 2; *β* = 0.12, *p* < 0.01; **H9**) were significantly related to affect dysregulation. Finally, the effect of dissociation on affect dysregulation was reduced after controlling for impulsiveness, compulsiveness, and alexithymia (path *c′* in Figure 2; *β* = 0.09, *p* < 0.05), albeit remaining significant, suggesting a partial mediation (**H10**): *R^2^* = 0.545, *F*(4, 425) = 148.503, *p* < 0.001. The bootstrapping technique confirmed the significance of the indirect effect: Boot LLCI = 0.110—Boot ULCI = 0.176.

All the models’ effects indices are displayed in Table 5.

## 4. Discussion

Addiction is a complex disorder affecting the functioning of the brain and body. Many approaches to the different psychological constructs underlying addiction have been taken in research, giving rise to interpretative theories and models (see Shafiee, Razaghi, & Vedadhir [38] for a review). Given this framework, the present study aimed to enrich and empirically test the Comprehensive Model of Addiction (CMA) [5] in a large sample of addicted individuals by defining the relationship between the factors that may contribute to the vulnerability and maintenance of the disorder.

Concerning the vulnerability model, all the hypotheses were supported: the results of the present study showed that insecure attachment in addicted individuals is linked to dissociation, with the mediation of alexithymia and complex trauma. These findings enrich and are in line with the pre-existing literature [39]. The first relational experiences of individuals provide the basis on which all subsequent adaptations will be built [40]: attachment is indeed associated with the development of emotional skills and the consequent functioning of the individual in the environment [41]. The lack of adequate interactive regulation skills in the relationship with the caregiver may affect the ability to mentalize [42] and transform emotional experiences into complex feelings [43]: the psychological distress of addicted individuals is not “*encoded in words*” [44] (p. 206) but is expressed in perceptual–action–affect responses [44,45]. Therefore, the mediating role of alexithymia in a vulnerability system acquires increased meaning, as the difficulty identifying and describing feelings and the tendency to display an externally-oriented thought impair the integration of mental states [46] and favor the development of addictive disorders [22,47,48,49]. In parallel, the results of this study also highlighted the impact of complex trauma, which was not identified as a relevant mediating factor among gamblers in the study of Gori and colleagues [5] but was supported for addiction in general by previous research [16,22]. Adverse childhood experiences favor increased sensitivity to subsequent distressing events [13]: under this condition, traumatic experiences may generate maladaptive defenses against painful events, facilitating the onset of dissociative symptoms and the search for psychological numbness to cope with distressing emotions [23,50]. Thus, it is understandable that dissociation (output in the vulnerability model) can facilitate the tendency to addictive behaviors [23,51,52], but it can also be seen as a key aspect in its maintenance (e.g., [53]). Dissociation might be conceived in the context of addictive behaviors as a defense against painful feelings that the individuals cannot manage functionally; accordingly, they might try to alleviate psychic pain by intensely absorbing, impulsively, compulsively, and obsessively, into the use of a substance or repeated behavior. Thus, altered states of consciousness are searched for first as a defense, but then they push the individual to live further in a constant condition of absorption and dissociation, which perpetuates dependence [22]. As Gold [54] (p. 1982) stated, “*every human problem has an attempt to solve a problem*”: indeed, in the short term, addictive behaviors can reduce or control suffering, but this strategy also makes it unlikely the development of adequate psychological skills for emotion regulation that are needed to cope with the challenges of life [55]. This will result in a vicious circle that will make addiction an increasingly necessary behavior.

Indeed, the second model confirmed the link between dissociation and affect dysregulation, both directly and indirectly, empirically supporting all the hypotheses concerning the maintenance model. Specifically, the indirect path involved impulsiveness and compulsiveness in influencing obsessiveness, and all of these, which are the core components of craving [5], showed an effect on affect dysregulation. Impulsiveness and compulsiveness, in line with Hollander [56], can be seen as two extremes of a continuum indicating on one side the uncontrolled search for pleasure (impulsivity and positive reinforcement) and on the other side the avoidance of pain typical of withdrawal symptoms (compulsion and negative reinforcement). This dynamic would generate both positive and negative memories (pleasure and displeasure) related to the behavior, which manifest themselves with obsessive, intrusive thoughts, especially in the presence of external or internal triggers, thus maintaining addiction. Furthermore, the model also supported the role of emotional dysregulation and emotional instability in favoring the motivational drive to addictive behavior and, thus, perpetuating the problem [57]. Hence, the maintenance of addiction appears to be strictly influenced by a dissociative push to implement impulsive and compulsive problematic behavior to avoid painful emotions. This makes the “object” of addiction (alcohol, gambling, gaming, etc.) the protagonist of the individual’s thoughts, which in turn further feeds dysregulated affect states.

This study comes with some limitations. First, the cross-sectional design implies caution in interpreting the causal links between the variables in the hypothesized models. A longitudinal approach could help to give further evidence in this regard. Furthermore, since the aim of this research was to test a comprehensive model of addiction, no discrimination has been made between the various kinds of addictive behaviors: this does not allow us to provide definitive conclusions about vulnerability and maintenance factors in specific addictive conditions. Considerations for future research include the possibility of analyzing the impact of the examined variables in different and specific kinds of addictive disorders, as has been done for pathological gambling [5]. Moreover, most study participants declared themselves to be unemployed and single. Since these characteristics may have played a role in the vulnerability/maintenance of addiction, future research is needed to replicate the results in samples with different demographic features. Consistently, the socioeconomic status of the participants was not explored in this study. Previous research showed that individuals with lower income were more likely to report having problems related to their substance abuse compared to individuals with higher income [58]. Therefore, the exploration of this aspect could be an interesting challenge for future research. Finally, adult attachment was assessed with a self-report scale [29]. Although this measure demonstrated excellent psychometric properties (see Justo-Núñez and colleagues [59] for a review), future research should replicate these results also using other instruments [60,61,62,63].

## 5. Conclusions

This study expands and integrates the previous theoretical evidence through an in-depth analysis and application of a Comprehensive Model of Addiction (CMA) in a large clinical sample of individuals diagnosed with substance use disorder. The findings provide a further contribution to the understanding of the specific factors that may be involved in the vulnerability to addictive disorders and their maintenance. The integration of this information with other research evidence (for example, those relating to the understanding of addiction severity [64]) can serve for the development of tailored preventive, clinical, and therapeutic interventions that are sensitive to the specific psychological needs and vulnerability of individuals with substance use disorder.

## Figures and Tables

**Table 1:** Demographic characteristics of the sample.

Characteristics		Sample
*Sex (%)*		
	Males	77.1
	Females	21.7
*Age (M, SD)*		36.3 ± 12.2
*Marital Status (%)*		
	Single	57.9
	Married	23.3
	Cohabiting	5.6
	Separated	6.7
	Divorced	4.9
	Widowed	1.4
	Missing Values	0.2
*Education (%)*		
	Elementary school (5 years)	5.1
	Middle School diploma (8 years)	38.4
	High School diploma (13 years)	42.3
	Bachelor’s degree (16 years)	5.1
	Master’s degree (18 years)	6.0
	Post-Lauream Specialization (22 years)	2.3
	Missing values	0.7
*Professional Condition (%)*		
	Unemployed	34.2
	Looking for the first job	2.8
	Entrepreneur	5.3
	Employee	15.6
	Artisan	4.9
	Trader	2.3
	Armed forces	0.5
	Housewife	1.9
	Student	13.7
	Retired	4.7
	Other	13.3
	Missing values	0.9

**Table 2:** Correlation matrix.

	1	2	3	4	5	6	7	8	9	10	11	12	13	14
(1) Secure attachment style	-	−0.109 *	−0.127 **	−0.208 **	−0.378 **	**−0.060**	**−0.087**	−0.287 **	−0.272 **	−0.116 **	−0.139 **	−0.177 **	−0.112 *	−0.211 **
(2) Preoccupied attachment style		-	**0.007**	0.355 **	0.376 **	0.255 **	0.340 **	0.658 **	0.480 **	0.345 **	0.203 **	0.367 **	0.372 **	0.451 **
(3) Avoidant attachment style			-	0.198 **	0.149 **	0.149 **	0.170 **	**0.081**	0.111*	0.154 **	0.131 **	0.193 **	0.142 **	0.177 **
(4) Unresolved attachment style				-	0.286 **	0.355 **	0.390 **	0.342 **	0.310 **	0.352 **	0.208 **	0.311 **	0.340 **	0.335 **
(5) Alexithymia					-	0.099 *	0.425 **	0.439 **	0.579 **	0.476 **	0.119 **	0.424 **	0.364 **	0.498 **
(6) Traumatic experiences						-	0.275 **	0.284 **	0.233 **	0.270 **	0.565 **	0.285 **	0.222 **	0.253 **
(7) Dissociation							-	0.353 **	0.417 **	0.591 **	0.232 **	0.409 **	0.454 **	0.432 **
(8) Separation anxiety								-	0.650 **	0.455 **	0.319 **	0.466 **	0.448 **	0.590 **
(9) Affect dysregulation									-	0.523 **	0.285 **	0.621 **	0.567 **	0.706 **
(10) Somatoform and psychological dissociation										-	0.242 **	0.496 **	0.531 **	0.524 **
(11) Childhood traumatic experiences											-	0.251 **	0.229 **	0.272 **
(12) Impulse dyscontrol												-	0.577 **	0.710 **
(13) Compulsive behavior and ritualization													-	0.659 **
(14) Obsessive thoughts														-

**Note**: bold values indicate significant *p*-values; **. Correlation is significant at the 0.01 level (2-tailed). *. Correlation is significant at the 0.05 level (2-tailed). *Secure attachment style* (PTI-ASS); *Preoccupied attachment style* (PTI-ASS); *Avoidant attachment style* (PTI-ASS); *Unresolved attachment style* (PTI-ASS); *Twenty-Items Toronto Alexithymia* (TAS20); *Traumatic Experiences Checklist* (TEC); *Dissociative Experiences Scale-II* (DES-II); *Barratt Impulsiveness Scale 11* (BIS11); *Separation anxiety* (ABQ); *Affect dysregulation* (ABQ); *Somatoform and psychological dissociation* (ABQ); *Childhood traumatic experiences* (ABQ); *Impulse Dyscontrol* (ABQ); *Compulsive behavior and ritualization* (ABQ); *Obsessive thoughts* (ABQ).

**Table 3:** Models’ Coefficients for the vulnerability model: a parallel mediation.

Antecedent		Consequent													
		M1					M2					Y			
		b	SE	*p*	95% CI		b	SE	*p*	95% CI		b	SE	*p*	95% CI
X	*a*_1_	18,527	2.181	<0.001	14.242, 22.811	*a_2_*	1.270	0.190	<0.001	0.896, 1.644	*c′*	0.971	0.173	<0.001	0.631, 1.311
M1		-	-	-	-		-	-	-	-	*b*_1_	0.012	0.003	<0.001	0.006, 0.019
M2		-	-	-	-		-	-	-	-	*b*_2_	0.315	0.037	<0.001	0.244, 0.387
Constant	*i_M_*_1_	30.719	18.156	0.091	−4.953, 66.391	*i_M2_*	39.832	1.545	<0.001	36.718, 42.946	*i_Y_*	−11.834	1.953	<0.001	−15.670, −7.998
		*R^2^ =* 0.126					*R^2^* = 0.082					*R^2^* = 0.281			
		*F*(1, 428) = 72.181, *p* < 0.001					*F*(1, 428) = 44.520, *p* < 0.001					*F*(3, 426) = 64.878, *p* < 0.001			

**Note***:* X = insecure attachment (PTI unresolved attachment style scale); M1 = complex trauma (TEC); M2 = Alexithymia (TAS-20); Y = Dissociation (DES-II).

**Table 4:** Models’ coefficients for the maintenance model: a mixed serial–parallel mediation.

Antecedent		Consequent																		
		M1					M2					M3					Y			
		b	SE	*p*	95% CI		b	SE	*p*	95% CI		b	SE	*p*	95% CI		b	SE	*p*	95% CI
X	*a*_1_	0.176	0.018	<0.001	0.141, 0.210	*a*_2_	0.178	0.016	<0.001	0.148, 0.209	*a*_3_	0.037	0.015	<0.05	0.007, 0.067	*c′*	0.038	0.001	<0.05	0.009, 0.067
M1		-	-	-			-	-	-		*b*_1_	0.521	0.039	<0.001	0.445, 0.596	*b*_3_	0.195	0.044	<0.001	0.109, 0.281
M2		-	-	-			-	-	-		*b*_2_	0.414	0.043	<0.001	0.329, 0.499	*b*_4_	0.125	0.046	<0.01	0.035, 0.214
M3		-	-	-			-	-	-			-	-	-	-	*b*_5_	0.409	0.044	<0.001	0.324, 0.495
Constant	*i_M_*	8.084	0.321	<0.001	7.454, 8.714	*i_M_*_2_	5.687	0.287	<0.001	5.124, 6.250	*i_M_*_3_	2.404	0.377	<0.001	1.663, 3.144	*i_Y_*	3.354	0.381	<0.001	2.606, 4.102
		*R^2^* = 0.167, *F*(1, 428) = 100.390, *p* < 0.001					*R^2^* = 0.206, *F*(1, 428) = 129.465, *p* < 0.001					*R^2^* = 0.602, *F*(3, 426) = 251.129, *p* < 0.001					*R^2^* = 0.545, *F*(4, 425) = 148.503, *p* < 0.001			

**Note**: X = dissociation (DES-II); M1 = impulsiveness (7DAS impulse dyscontrol scale); M2 = compulsiveness (7DAS compulsive behavior and ritualization scale); M3 = obsessiveness (7DAS obsessive thoughts scale); Y = affect dysregulation (7DAS affect dysregulation scale).

**Table 5:** Models effect indices.

Model	Total Effect	Direct Effect	Indirect Effect	Partial Standardized Indirect Effect	Completely Standardized Indirect Effect	Bootstrapping 95% CI
Vulnerability model	1.600	0.971	0.629	0.051	0.154	(0.384, 0.918)
Maintenance model	0.177	0.038	0.139	0.027	0.328	(0.110, 0.176)

## Data Availability

The data presented in this study are available on request from the corresponding author. The data are not publicly available due to privacy reasons.
